# Amelanotic Malignant Melanoma With Atypical Divergent Neuroendocrine Differentiation: A Report of an Unusual and Rare Case of Anorectal Bleeding

**DOI:** 10.7759/cureus.66905

**Published:** 2024-08-14

**Authors:** Shamiliprabha MG, Anand CD, Supriya Verma, Nivethitha S, Jaison J John

**Affiliations:** 1 Department of Pathology, Sri Ramaswamy Memorial (SRM) Medical College Hospital and Research Centre, SRM Institute of Science and Technology (SRMIST), Chengalpattu, IND

**Keywords:** poorly differentiated malignancy, anorectal amelanotic melanoma with neuroendocrine differentiation, amelanotic melanoma, malignant melanoma, divergent differentiation

## Abstract

Melanocytes located between the anal transition zone and the dentate line of the anal canal can give rise to the uncommon malignant tumor known as anal melanoma. It has a fast-paced clinical course and can masquerade as several common anorectal symptoms, such as hemorrhoids or rectal ulcers. In melanoma, divergent differentiation is a very uncommon phenomenon. The diagnosis of melanoma is difficult with histopathology sections alone (hematoxylin and eosin, H&E). Special stains and ancillary immunohistochemistry investigations are useful in these situations. A 60-year-old female patient presented to the surgical outpatient department with complaints of anorectal bleeding. After clinical evaluation, a growth in the anorectal region was identified, and a biopsy was taken from the growth. Histopathological and subsequent immunohistochemical analysis of the biopsy material was done at the Department of Pathology. A diagnosis of amelanotic melanoma with atypical and divergent neuroendocrine differentiation involving the anorectal region was rendered. Histologically, this tumor showed extremely pleomorphic polygonal to elongated spindle cells that co-expressed neuroendocrine markers and were positive for S100, HMB-45, and Melan-A. This case presented many diagnostic challenges at both the histomorphological level and the immunohistochemical expression profile analysis. We will go into great depth regarding the diagnostic challenges in this instance and provide an outline of our approach. The immunohistochemical and prognostic importance of this case will also be covered.

## Introduction

Anorectal melanoma occurs more commonly in elderly females and constitutes only 0.1%-4.6% of anal tumors [1-3]. It arises from the malignant transformation of melanocytes situated between the anal transition zone and the dentate line [4]. These melanocytes are embryologically descended from the neural crest [5].

Anorectal melanoma is a mucosal melanoma and the third most common site for melanoma after the skin and retina [6]. Ocular and mucosal melanomas usually have a poor prognosis [7].

The cytomorphology of atypical melanocytes varies from round to oval/spindle/epithelioid/plasmacytoid/lymphocyte-like appearance. The characteristic feature of melanoma is the presence of a prominent eosinophilic nucleolus and abundant fine melanin pigment in the cytoplasm [8-10]. One of the differential diagnoses to be considered when encountering a poorly differentiated malignant tumor on histopathology, especially from growths in the anorectal region, is amelanotic malignant melanoma [11]. Immunohistochemical analysis is critical in such cases to establish a diagnosis of malignant melanoma. Melanomas usually show diffuse positivity for S-100 (88% sensitivity and 70% specificity) and HMB-45 (92% sensitivity and 97% specificity) immunohistochemical (IHC) stains. In addition, Melan-A has a higher sensitivity of 95% and specificity of 97% in melanomas. Hence, HMB-45 and Melan-A are considered more melanocyte-specific [12].

## Case presentation

**Figure 1:**
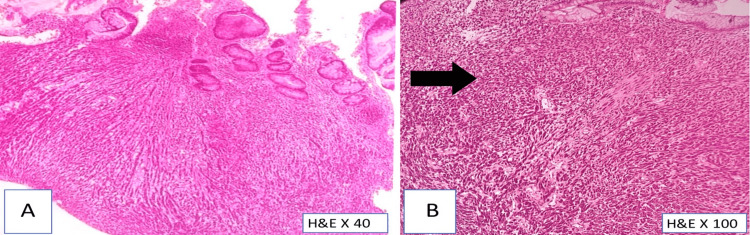
H&E image (A) Anorectal mucosa infiltrated by tumor cells; (B) tumor cells (black arrow) arranged as cords, trabeculae, and sheets with few remnants of colonic epithelium H&E: hematoxylin and eosin

A 60-year-old female patient presented to the surgical outpatient department with complaints of anorectal bleeding, loose stools, and pain in the perianal region for the past six months. A colonoscopy revealed nodular ulcerative growth at 7 o'clock, 2 cm from the lower edge of the anal canal. A biopsy was taken and sent for histopathological examination. We received the biopsy in multiple small gray-brown tissue fragments, measuring 0.3 cm^3^ (cubic centimeter) in total aggregations. Microscopically, the anorectal mucosa is infiltrated by tumor cells arranged in cords, trabeculae, and sheets (Figure 1).

**Figure 2:**
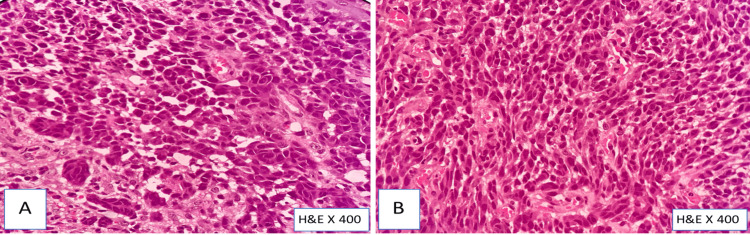
H&E image (A) The tumor cells are arranged in sheets with round to oval, elongated, and spindle-shaped cells with a high nuclear-to-cytoplasmic ratio; (B) the tumor cells show moderate to marked nuclear pleomorphism, hyperchromatism with mild to moderate cytoplasm, and inconspicuous nucleoli. No melanin pigment was noted in the sections studied H&E: hematoxylin and eosin

**Figure 3:**
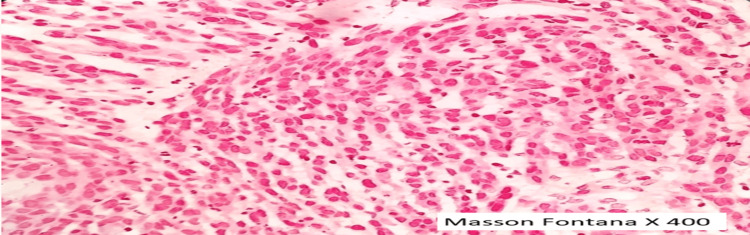
Special stain—Masson Fontana Tumor cells are negative for the special stain for melanin—Masson Fontana

Individual tumor cells were highly pleomorphic, polygonal to elongated/spindle-shaped, with a high nuclear-to-cytoplasmic ratio, moderate eosinophilic cytoplasm, a round-oval to irregular hyperchromatic nucleus, inconspicuous nucleoli, and a 4-5/10 high power field of atypical mitosis (Figure 2). The tumor cells were negative for Masson Fontana (histochemical stain for melanin) (Figure 3). Based on these findings, the most suitable diagnosis of poorly differentiated malignancy was made, and we advised an IHC panel for confirmation of the cell of origin (Pan CK, vimentin, CK20, synaptophysin, and HMB-45). 

**Figure 4:**
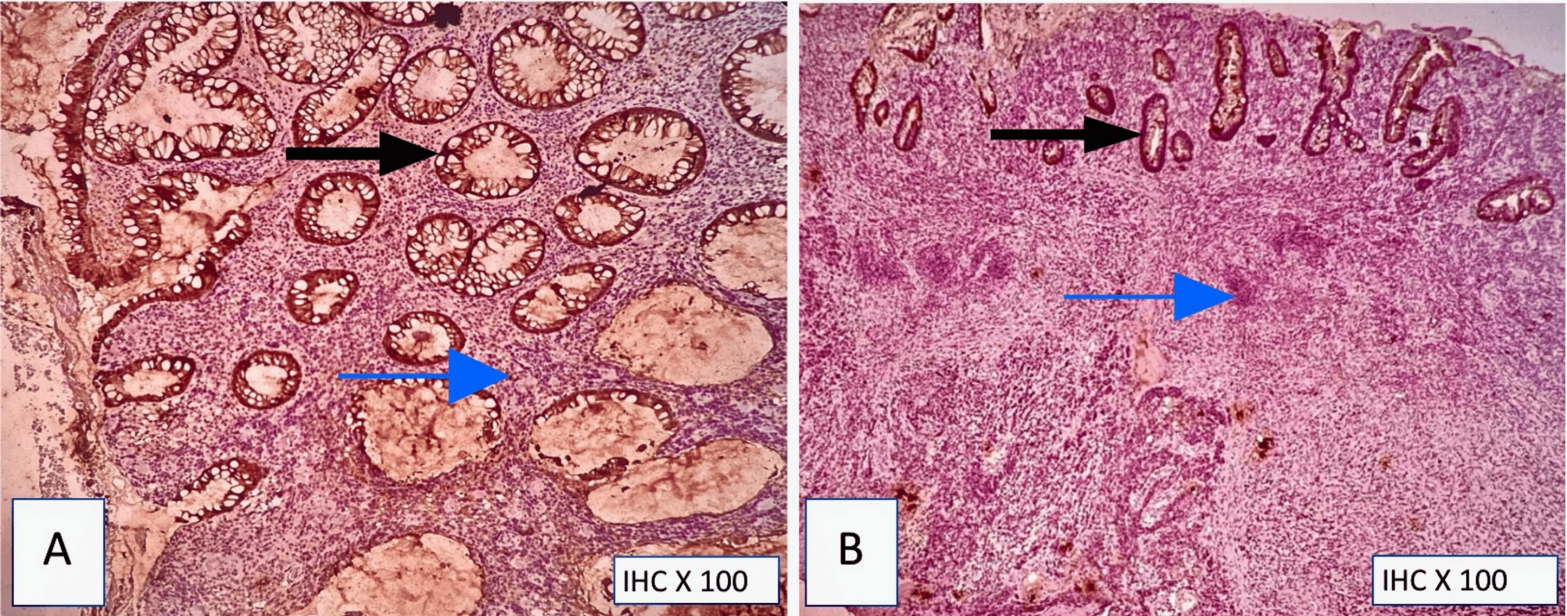
Pan-CK and CK-20 immunohistochemistry (A) PAN-CK: negative in tumor cells and positive in benign colonic epithelial cells. The blue arrow points to tumor cells, and the black arrow points to benign colonic epithelium; (B) CK-20: negative in tumor cells and positive in benign colonic epithelial cells. The blue arrow points to tumor cells, and the black arrow points to benign colonic epithelium

**Figure 5:**
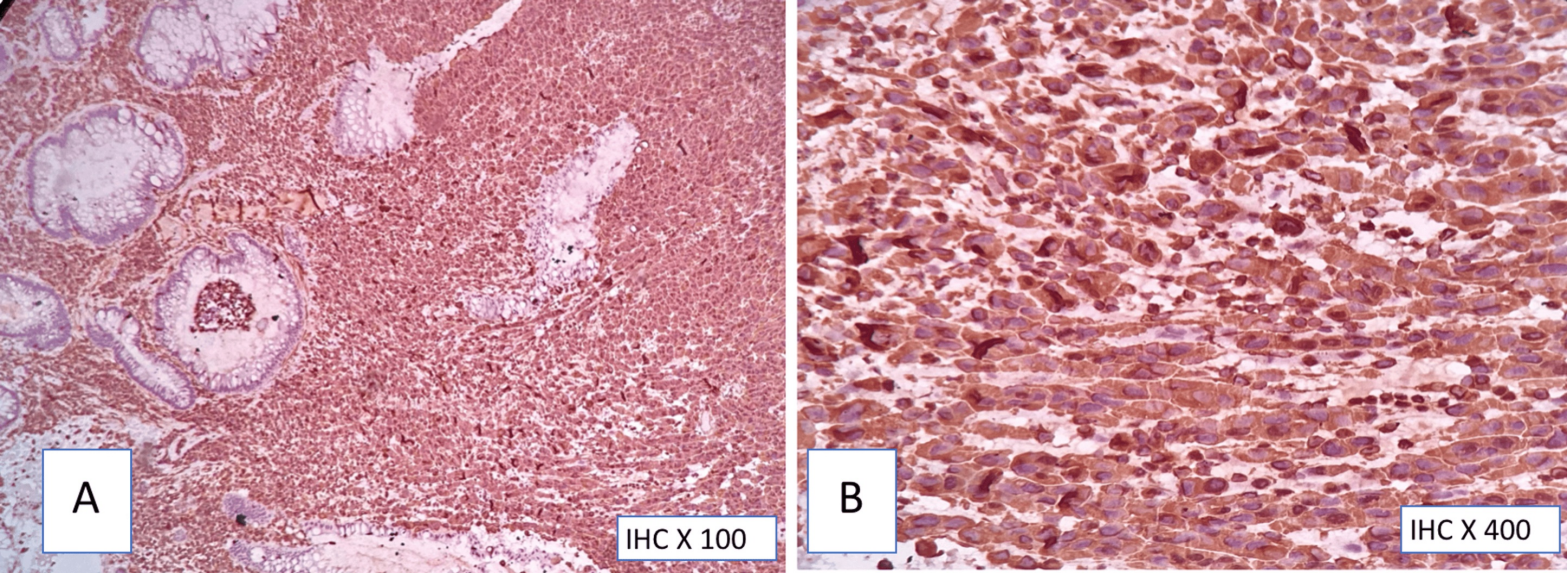
Vimentin immunohistochemistry (A,B) Vimentin shows diffuse, strong cytoplasmic positivity in tumor cells

**Figure 6:**
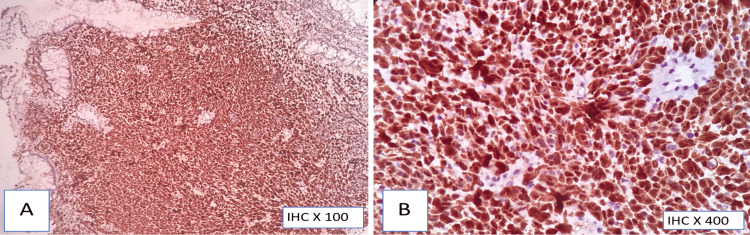
S100 immunohistochemistry (A,B) Diffuse strong cytoplasmic and nuclear positivity in tumor cells

**Figure 7:**
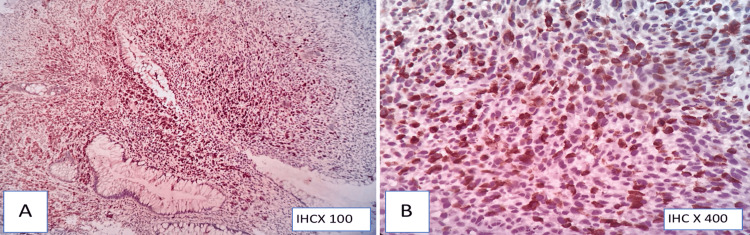
HMB-45 immunohistochemistry (A,B) Strong cytoplasmic positivity (70% of tumor cells)

**Figure 8:**
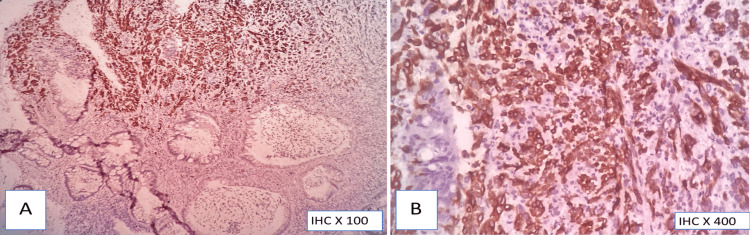
Melan-A/MART-1 immunohistochemistry (A,B) Cytoplasmic positivity in tumor cells

**Figure 9:**
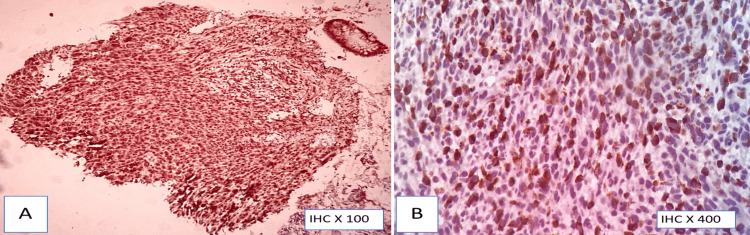
Synaptophysin immunohistochemistry (A,B) Diffuse strong cytoplasmic positivity in tumor cells

Immunohistochemical stains showed positivity in tumor cells for S-100 (Figure 6), HMB-45 (Figure 7), and Melan-A (Figure 8). Diffuse, strong positivity for vimentin was also noted (Figure 5). The tumor cells were also positive for synaptophysin (Figure 9) and were negative for PAN-CK and CK-20 (Figure 4).

Therefore, the final diagnosis of primary malignant melanoma (amelanotic type) with neuroendocrine differentiation was established based on histological features and the co-expression of melanocytic and neuroendocrine markers such as Melan-A, HMB-45, and synaptophysin. The patient was further evaluated by an oncologist, who determined that there was widespread and extensive distant metastasis to the lungs, liver, and skeletal system. The patient was discharged as per the patient and family's request for palliative and alternative therapy, and the current status is not known. The patient's family is not responding to calls from the hospital (lost to follow-up).

## Discussion

Anal melanoma is a cancerous condition that arises from melanocytes present between the anoderm and dentate line [4]. Melanoma of the anorectum is historically quite uncommon and is the second most common site for mucosal melanomas [13], occurring in the sixth to seventh decade with a slight female predilection [14]. Anorectal melanoma is very aggressive, with too early metastasis and a high level of mortality [5]. The most typical indications and symptoms of anal melanoma include rectal bleeding, mass, and pain [6]. Anorectal melanoma lesions are best evaluated by a colonoscopy-guided biopsy [13]. In general, melanomas range in size from 1 to >6 cm and are typically polypoid [15-17]. Up to 95% of cases have some form of ulceration. Bleeding may be incorrectly recognized as hemorrhoids, delaying the diagnosis [6]. Furthermore, an unanticipated diagnosis of anal melanoma may be made following the removal of a benign-appearing "hemorrhoid" or "skin tag" [6]. Upon microscopic investigation, only approximately 20% of lesions in the anal canal are found to be amelanotic [18], despite the fact that up to 80% of lesions may not exhibit any noticeable pigmentation upon clinical assessment [19]. Tumor thickness and the disease stage at the time of diagnosis are prognostic variables [1,20].

Considering the morphological diversity, amelanotic melanoma may be mistaken for poorly differentiated squamous cell carcinoma, lymphoma, or sarcoma, as histologically all are made up mostly of small to medium-sized, round to oval cells that are organized in solid sheets inside a vascular stroma [14,21].

The gold standard for diagnosing melanoma in the H&E section is the presence of intracellular melanin in malignant cells [22]. Special stains such as Masson Fontana and melanin bleach can be utilized to confirm melanin pigment [23].

Characteristic features of melanoma

In our case, the melanin pigment and the presence of a prominent eosinophilic nucleolus were absent, further complicating the distinction between melanoma and other poorly differentiated malignancies.

Immunohistochemistry is used to diagnose melanoma in amelanotic melanoma cases [23]. Tumor cells in melanoma show positivity for S-100, HMB-45, vimentin, Melan-A, tyrosinase, and microphthalmia-associated transcription factor (MITF). The S100 IHC stain, the most frequently used screening tool for melanocytic differentiation, is highly sensitive but lacks specificity [23]. Histologically, melanoma demonstrates a high degree of divergent differentiation, such as fibroblastic, myofibroblastic, Schwannian, perineurial, smooth muscle, rhabdomyosarcomatous, osteocartilaginous, ganglionic, ganglioneuroblastic, neuroendocrine, and epithelial [24]. This behavior has been referred to by some authors as a melanoma's metaplastic transformation [25].

Mechanisms of divergent differentiation

Once inside a tumor mass, neoplastic cells can change their differentiation according to external influences; this process, more recently termed neo- or trans-differentiation, eventually results in novel phenotypes [26,27]. Neuroendocrine differentiation and other subtypes of divergent differentiation in melanoma are highly unusual, and their significance is unclear in terms of prognosis and treatment [14,24].

Diagnostic pitfalls

Non-specificity of symptoms, lesions frequently manifesting as polyps or hemorrhoids, varied histomorphology, lack of melanin pigment in one-third of cases, abnormal expression of non-melanocytic IHC markers more frequently, and loss of one or more melanocytic markers are among the diagnostic traps associated with melanoma [13,21]. Pathologists are expected to have a significantly reduced likelihood of misdiagnosing uncommon cases where they appear as non-melanocytic tumors with divergent differentiation if they are aware of these potentially serious problems.

For anorectal melanoma, surgical removal is typically regarded as the first line of treatment. Chemotherapy (Dartmouth regimen) and radiotherapy are also effective. PDL-1 blockers, such as pembrolizumab, are now the standard treatment therapy for melanoma [13].

## Conclusions

For any poorly differentiated malignancy, melanoma should be considered a differential. Similar to our case, melanomas can exhibit divergent differentiation, just like metaplastic carcinomas. This is undoubtedly a rare occurrence that, when it does occur, can be overlooked by pathologists and result in confusion regarding the diagnosis. An accurate final diagnosis and prognostication can be achieved by using a wide and properly selected panel of IHC to shed light on the type of differentiation present in these tumors. To further our understanding of this uncommon disease, we add a new instance of amelanotic malignant melanoma with neuroendocrine differentiation to the body of literature, suggesting that pathologists and clinicians should be aware of its possibility at this site and all unusual, atypical anal masses to be biopsied and malignancy to be ruled out. Nevertheless, the therapeutic and prognostic effects of these divergent differentiation variants of melanoma remain uncertain, and additional comprehensive investigations are necessary to address this matter.
